# The Nurse-Based Age Independent Intervention to Limit Evolution of Disease After Acute Coronary Syndrome (NAILED ACS) Risk Factor Trial: Protocol for a Randomized Controlled Trial

**DOI:** 10.2196/resprot.3466

**Published:** 2014-08-15

**Authors:** Thomas Mooe, Fredrik Björklund, Anna Graipe, Daniel Huber, Stina Jakobsson, Ulf Kajermo, Anna Strömvall, Anders Ulvenstam

**Affiliations:** ^1^Department of Public Health and Clinical MedicineUmeå UniversityÖstersundSweden; ^2^Section of CardiologyDepartment of Internal MedicineÖstersund HospitalÖstersundSweden; ^3^Department of CardiologySkaraborg HospitalSkövdeSweden

**Keywords:** acute coronary syndrome, myocardial infarction, secondary prevention, cardiovascular disease, randomized controlled trial

## Abstract

**Background:**

Secondary prevention after acute coronary syndrome (ACS) is essential to reduce morbidity and mortality, but related studies have been fairly small or performed as clinical trials with non-representative patient selection. Long-term follow-up data are also minimal. A nurse-led follow-up for risk factor improvement may be effective, but the evidence is limited.

**Objective:**

The aims of this study are to perform an adequately sized, nurse-led, long-term secondary preventive follow-up with inclusion of an unselected population of ACS patients. The focus will be on lipid and blood pressure control as well as tobacco use and physical activity.

**Methods:**

The study will consist of a randomized, controlled, long-term, population-based trial with two parallel groups. Patients will be included during the initial hospital stay. Important outcome variables are total cholesterol, low-density lipoprotein (LDL) cholesterol, and sitting systolic and diastolic blood pressure. Outcomes will be measured after 12, 24, and 36 months of follow-up. Trained nurses will manage the intervention group with the aim of achieving set treatment goals as soon as possible. The control group will receive usual care. At least 250 patients will be included in each group to reliably detect a difference in mean LDL of 0.5 mmol/L and in mean systolic blood pressure of 5 mmHg.

**Results:**

The study is ongoing and recruitment of participants will continue until December 31, 2014.

**Conclusions:**

This study will test the hypothesis that a nurse-led, long-term follow-up after an ACS with a focus on achieving treatment goals as soon as possible is an effective secondary preventive method. If proven effective, this method could be implemented in general practice at a low cost.

**Trial Registration:**

International Standard Randomized Controlled Trial Number (ISRCTN): 96595458; http://www.controlled-trials.com/ISRCTN96595458 (Archived by WebCite at http://www.webcitation.org/6RlyhYTYK).

## Introduction

### Background

Clinical trials have identified several treatments with proven secondary preventive efficacy after an acute coronary syndrome (ACS) [1]. Implementation of this knowledge, however, faces a number of obstacles and has been more difficult than anticipated [2] for a number of possible reasons. Among these, clinical trials typically include selected populations with lower age and less comorbidity compared with patients in routine clinical practice. In addition, trials generally are rigorously performed during a fairly short period of time, while the clinical environment is complex and difficult to control and the treatment perspective often spans many years. In secondary prevention these difficulties have led to failures in terms of treatment goals and consequently failure in risk reduction [3].

To simplify secondary preventive follow-up procedures while also retaining a high level of treatment adherence, different strategies of telephone support have been evaluated [4-6]. Despite rigorous protocols, ambitious patient information packages, and frequent patient support, the results in terms of reduction of risk factor levels have been modest [7,8]. To some extent, this outcome may be explained by improvements in general care against which intervention results are compared. However, the published secondary preventive trials using telephone support have several weaknesses, including a low proportion of included patients relative to the target population [5,6]. Other weaknesses are age limits, extensive protocols requiring an alert patient, recruitment from selective secondary prevention programs, and exclusion because of comorbidity. Furthermore, a rapid routine intended to allow for prescription changes for lipid and blood pressure control to achieve treatment goals as soon as possible has mostly not been used. Moreover, low-risk patients have been included with a 12-month mortality rate around 2% [7,8]. Thus, more research in this area is needed.

### Study Objective

To improve secondary prevention, we have designed a trial with several important components. First, all patients after an ACS will be considered for inclusion regardless of age, and this population-based design will include all residents in the county of Jämtland, Sweden. Second, a maximally simplified, nurse-based, telephone follow-up protocol will be used, which will minimize resources needed as well as patient exclusions and make the methodology easy to implement. Third, a routine for prompt medication adjustment will be used to reach set treatment goals as quickly as possible. Finally, a long-term follow-up is planned with risk factor evaluation after 12, 24, and 36 months.

### Hypothesis

We hypothesize that in ACS patients, this nurse-based, telephone follow-up will reduce risk factor levels more effectively than usual care.

## Methods

### Trial Design

This study is planned as a randomized, controlled trial with two parallel groups and an allocation ratio of 1:1.

### Ethical and Research Governance Approval

Ethics approval has been received from the Ethics Committee, Umea University (09-142M). This paper presents the design of the study according to CONSORT requirements [9].

### Participants

All patients living in the county of Jämtland, Sweden, who were hospitalized with a diagnosis of myocardial infarction or unstable angina will be assessed for inclusion. Östersund Hospital is the only hospital in the county, and all patients, those in terminal care excluded, with symptoms of a suspected ACS are referred for diagnostic evaluation. It is a rural catchment area with a population of approximately 125,000 inhabitants. A routine for identification of all patients in the hospital with a possible ACS has been established in previous studies, and during a 3-month test period, the study nurses identified all patients with a final ACS diagnosis. In the present study, an ACS was defined as myocardial infarction type 1 or unstable angina with electrocardiogram changes (ST depression or T wave changes) suggesting myocardial ischemia. All patients with the physical and mental capacity to communicate by telephone will be eligible, which means that those with deafness or dementia will be excluded. The other exclusion criteria are severe, often terminal, disease and participation in another ongoing trial.

### Interventions

All eligible patients will be informed about the study and asked to give written informed consent. They will receive standard information about ACS concerning pathophysiology and risk factors according to established clinical practice during their hospitalization. They will also be offered a follow-up visit to a cardiology nurse and an outpatient follow-up according to usual care.

A study nurse will contact patients randomized to the intervention group by phone 1 month after discharge. Before the call, a blood sample for lipids will be taken and a standardized blood pressure control performed. Blood pressure will be measured after 5 minutes in the sitting position and after 1 minute standing. A district nurse will perform the tests; for patients in the intervention group living close to the hospital, a study nurse will do the testing. Cardiac symptoms and self-reported adherence with medication will be recorded. During the call, the patient will be informed about the test results and whether a change in medication is necessary. Tobacco use, physical activity, and dietary habits will be discussed. Smoking cessation will be encouraged and supported. Physical activity of moderate intensity 30 minutes or more on most days of the week will be encouraged but also adjusted to the individual patient’s capacity. Dietary advice to reduce saturated fat and increase fruit and vegetable intake will be given. If the patient’s cholesterol or blood pressure values are above target, medication will be adjusted after contact from a study physician. Tests will be repeated within approximately 4 weeks, and further adjustments made if necessary until target values are reached or no further changes can be considered realistic. The same routine, with an Hb1C test added, will be applied after 12, 24, and 36 months. The patient’s motivation to follow instructions will be assessed.

The target values are systolic blood pressure <140/<90 mmHg, total cholesterol <4.5 mmol/L, and low-density lipoprotein (LDL) cholesterol <2.5 mmol/L (optionally <1.8 mmol/L in patients at very high risk, eg, who have diabetes) [10,11].

**Figure 1:**
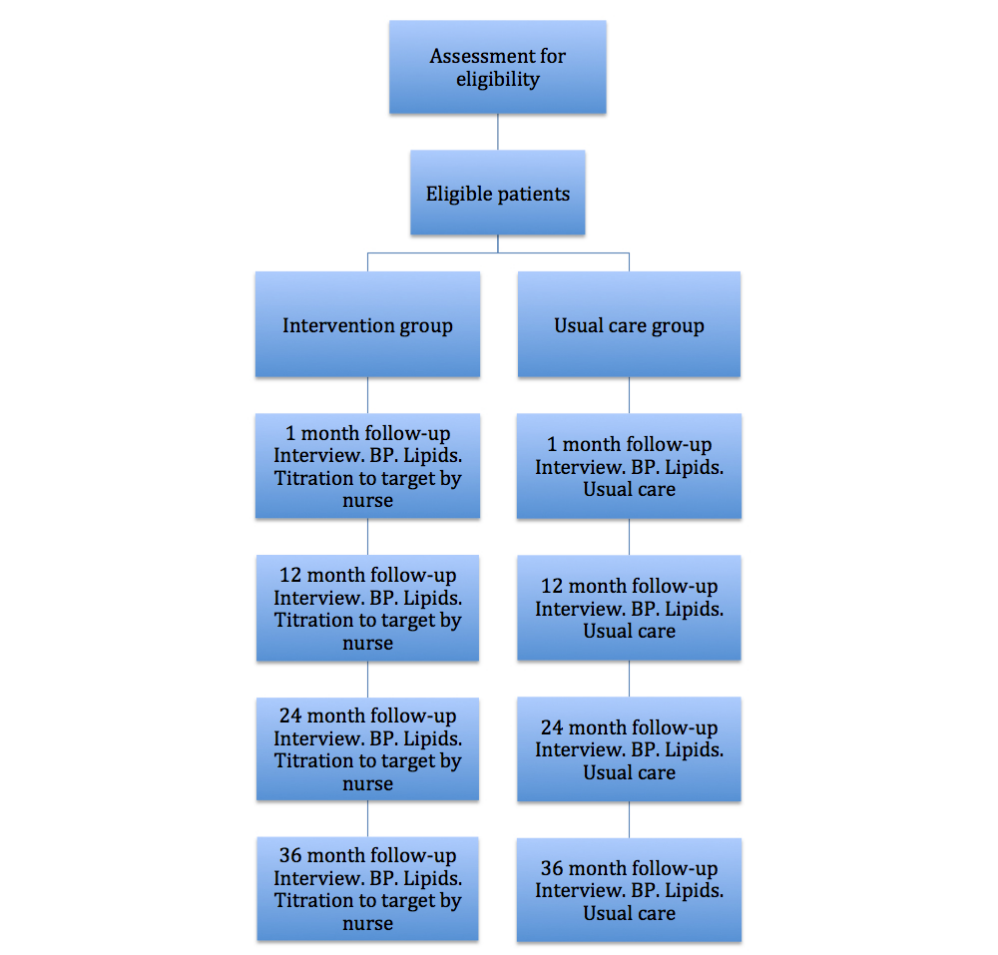
Study flow chart (BP=blood pressure).

Patients randomized to the usual care group will also be contacted by phone 1 month following discharge after blood pressure and lipid profile measurements. Cardiac symptoms, self-reported compliance, tobacco use, and physical activity will be recorded. The treating physician, usually a general practitioner, will provide all medical care and receive the test results (lipid profile and blood pressure), and no additional intervention will be given as a result of participation in the study. The scheduled controls of blood pressure and lipids in the study will be additive to the usual follow-up performed by the general practitioner. The same routine, with an Hb1C test added, will be applied after 12, 24, and 36 months (Figure 1). Participation in the study will otherwise not affect the standard of care in the usual care group.

The number of visits to the patient’s treating physician as well as visits to a nurse will be recorded to develop a picture of the number of medical assessments in the control and intervention groups.

### Outcomes

Outcomes will be measured after 12, 24, and 36 months of follow-up. Outcome variables are total cholesterol, LDL cholesterol, and sitting systolic and diastolic blood pressure as well as the proportion of patients achieving the set target for these variables. Standing systolic and diastolic blood pressure, smoking rates, the proportion of patients treated with different secondary preventive drugs, diabetes control by Hb1C, and change in body mass index (BMI) and physical activity will also be measured. The LDL value at 36 months will be analyzed as the primary outcome. Analyses of the secondary outcomes will be exploratory. Blood pressure measurements are standardized as described above, and LDL values are calculated from the serum concentrations of cholesterol and fasting triglycerides using the Friedewald formula. Smoking (yes/no) and physical activity (duration/week) are self reported. Deaths are available in the hospital records and will be recorded to detect any survival difference between groups.

### Sample Size

A difference between groups in mean LDL of 0.5 mmol/L and in mean systolic blood pressure of 5 mmHg is considered clinically relevant and requires study groups of approximately 200 participants to be reliably detected (alpha .05, two-tailed, power 80%). Study groups of at least 250 participants are planned to allow for drop-outs. This sample size is also adequate for detection of clinically meaningful group differences in smoking rates (10%), proportion reaching treatment goals (10%), change in BMI (1.0), and change in physical activity (10%, proportion in a given activity level), calculated with two-tailed alpha .05, power 80%.

### Randomization

The random allocation sequence will be computer generated in blocks of four and stratified for sex and type of ACS (unstable angina or myocardial infarction). A sealed, colored envelope will have a serial number on the outside and a folded sheet of paper inside with the group allocation written on it, which will be impossible to read from the outside. The study coordinators will enroll participants and assign them to interventions in order according to the serial number. The random allocation sequence will be computer generated by the study manager, who is not involved in the randomization process.

### Statistical Methods

Mean values of blood pressure and lipid variables in the intervention and control groups will be compared using the *t* test for independent groups. Proportions will be compared using the chi-square test. The primary analysis will be performed according to the intention-to-treat principle using a linear regression model adjusting for sex and type of ACS. The adjustment is made to reflect the stratified randomization process. Per protocol analyses will also be performed. Secondary outcomes will be analyzed using the primary analysis model when continuous, and a logistic regression model, adjusting for the same covariates, when outcomes are binary. To assess indications of differential treatment effects across subgroups (ie, age, sex, comorbidity, level of education, and social classification), tests for interaction will be performed, although this aim is secondary because the study is not powered for this particular purpose. Adjustment for relevant baseline covariates will be performed in additional exploratory analyses of primary and secondary outcomes to evaluate the effect of possible baseline imbalance. All tests will be two-sided, and a *P* value of <.05 will be considered significant.

## Results

The study is ongoing and recruitment of participants will continue until December 31, 2014.

## Discussion

### Summary

Different approaches have been used to recruit patients for secondary prevention studies after an ACS. Some have involved contacting patients participating in rehabilitation programs [5], and others have identified patients with a previous diagnosis of coronary artery disease [6]. To avoid selection bias, a population-based approach is preferable and is the only way to obtain a reliable estimate of the proportion of excluded patients. Enrollment of the patients immediately after an acute event is probably the best way to achieve a high participation rate. This study covers all patients in a Swedish county, who will be eligible for inclusion after an ACS, thus avoiding selection bias. Thus, the study population will include even very old and very sick ACS patients, who are usually not part of randomized ACS studies. Their ability to participate in secondary prevention programs and their tolerability of medication when attempting to achieve set treatment goals are of great interest because these factors could have significant consequences for health economics and morbidity and mortality rates. The immediate recruitment and the logistics for including each and every ACS patient will show the true proportion of possible participants in the secondary preventive program.

A randomized design is necessary to reliably evaluate the effect of the intervention. Some investigators use a prospective cohort design, which makes it difficult to draw conclusions in terms of efficacy, particularly if the drop-out rate is high [12]. The design of the present study means that patients in the usual care group will be contacted by the study nurses to collect data about risk factors. This step adds a certain interference and possibly improvement in usual care but cannot be avoided if the results of the two groups are to be compared. The standard of care that is achieved in the intervention and control group can be assessed by comparing lipid and blood pressure values as well as other risk factor results with the corresponding values in other ACS populations.

We use an associated study physician for the intervention group to obtain immediate decisions about more qualified treatment changes. Based on the results of previous studies, rapid treatment decisions by someone directly involved in the study are related to more effective risk factor control [13].

The duration of follow-up has varied substantially among studies [8,14]. A follow-up period of at least 12-24 months seems necessary to evaluate the intervention during a clinically relevant period of time.

Different treatment goals for lipids and blood pressure have been used in different trials, but an LDL goal of <2.5 mmol/L and a blood pressure goal of <140/<90 are common [8]. These goals correspond well to recent American Heart Association /American College of Cardiology Foundation guidelines [15]. An option to treat lipids more aggressively in very high-risk patients with an LDL goal <1.8 mmol/L is included but given a lower class of recommendation. An LDL goal of <1.8 mmol/L in patients at very high cardiovascular disease risk is recommended in the recent European Society of Cardiology guidelines [16]. However, hard endpoint data from clinical trials using defined treatment goals are still lacking.

Secondary prevention after an ACS needs to be improved and to include a larger proportion of the patients. Importantly, the treatment goals need to be maintained over the long term. Even in trials that largely rely on telephone follow-up, the proportion of the target population eventually included has so far been small [5,6,17].

### Conclusion

The present trial is designed to be simple and to require a minimum of health care resources while simultaneously giving patients an effective secondary prevention. The methodology can be widely generalized and makes the inclusion of a majority of the ACS population possible, regardless of the health care system.

## Abbreviations

ACS: acute coronary syndrome
BMI: body mass index
LDL: low-density lipoprotein
